# Baseline susceptibility of a wild strain of main vectors of leishmaniasis to WHO-recommended insecticides in southeastern Iran

**DOI:** 10.1186/s13071-022-05154-5

**Published:** 2022-01-31

**Authors:** Yaser Salim Abadi, Alireza Sanei-Dehkordi, Maryam Hakimi Parizi, Abass Aghaei Afshar, Iraj Sharifi, Mohammad Amin Gorouhi, Leila Shirani-Bidabadi, Ismaeil Alizadeh

**Affiliations:** 1 Department of Health Services and Health Promotion, School of Health, Rafsanjan University of Medical Sciences, Rafsanjan, Iran; 2 Department of Medical Entomology and Vector Control, School of Health, Hormozgan University of Medical Sciences, Bandar Abbas, Iran; 3 Leishmaniasis Research Center, Kerman University of Medical Sciences, Kerman, Iran; 4 Research Center of Tropical and Infectious Diseases, Kerman University of Medical Sciences, Kerman, Iran; 5 Department of Vector Biology and Control, Faculty of Public Health, Kerman University of Medical Sciences, Kerman, Iran

**Keywords:** Susceptibility test, *Phlebotomus sergenti*, *Ph. papatasi*, *Ph. alexandri*, Leishmaniasis

## Abstract

**Background:**

In Iran, both cutaneous leishmaniases (CL) and visceral leishmaniases (VL) are endemic, recording one of the 10 highest CL prevalence in the world. Parasites are transmitted by the bite of infected *Phlebotomus* sand fly females. Several sand fly species have been identified as vectors in the studied region of Kerman province. Residual spraying to control adult sand flies, is the only way to decrease the spreading of the diseases but, following control treatment against malaria vectors in endemic areas in Iran, resistance or tolerance to insecticides emerged in some sand fly species. The objective of this study was to survey insecticides susceptibility levels of 3 vector species in wild sand fly populations in different foci of the diseases in Kerman province. *Ph. sergenti*, and *Ph. papatasi* respectively vectors of anthroponotic and zoonotic cutaneous leishmaniases and for the first time *Ph. alexandri* one of the anthroponotic visceral leishmaniases vector were tested against: deltamethrin 0.05%, malathion 5%, dichloro-diphenyl-trichloroethane (DDT) 4%.

**Materials and methods:**

In leishmaniases endemic areas species specific sand fly sites were selected in Kerman province, and specimens were collected by manual aspirators at different time intervals during the spring and summer 2019. All the susceptibility tests were performed according to the WHO tube test recommended procedure.

**Results:**

Twenty five blood-fed female sand flies from the region's prevalent species were used in each pooled test replicates. All wild specimens died within 60 min of exposure to DDT 4%, malathion 5%, and deltamethrin 0.05%, but the mortality rate for *Ph. papatasi* exposed to malathion and DDT was 91.6% and 66.6%, respectively.

**Conclusion:**

According to current study results, *Ph. sergenti* and *Ph. alexandri* are highly susceptible to all the evaluated insecticides in the study areas. However, *Ph. papatasi* was susceptible to deltamethrin (100% mortality), possibly resistant or tolerant to malathion (91.6% mortality), and confirmed to be resistant to DDT (66.6% mortality).

**Graphical Abstract:**

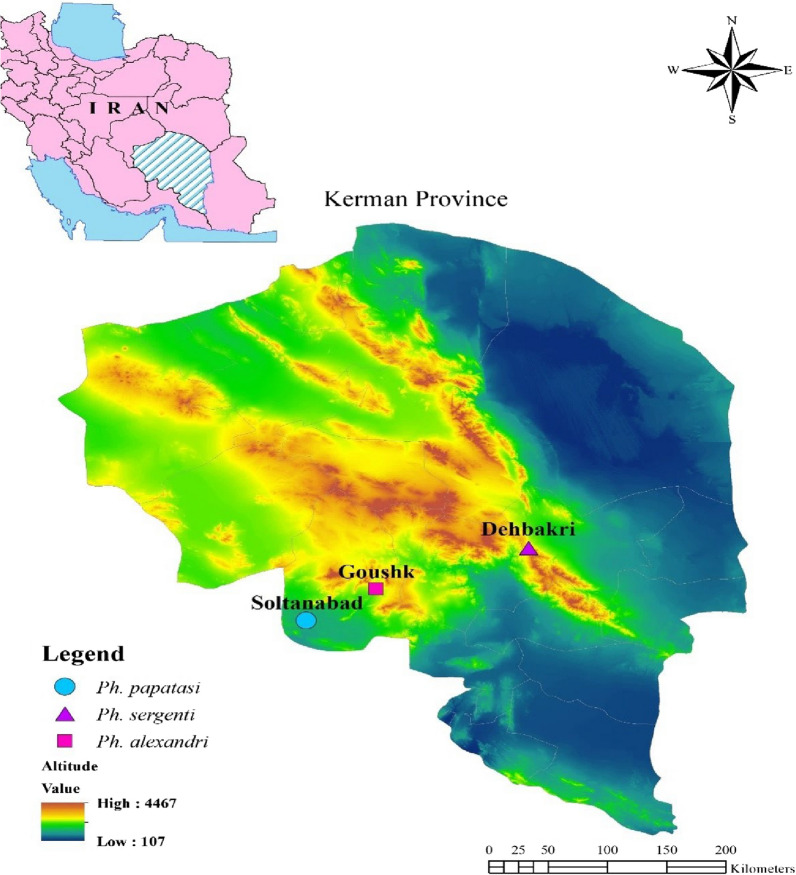

## Background

Two types of leishmaniasis, cutaneous leishmaniasis (CL) and visceral leishmaniasis (VL), are endemic in Iran. Iran ranks among the 10 countries with the highest prevalence of CL worldwide, which is caused by *Leishmania tropica* and *Leishmania major* (Kinetoplastida: Trypanosomatidae) that are transmitted by the bite of infected female sand flies *Phlebotomus sergenti* and *Phlebotomus papatasi* (Diptera: Psychodidae), respectively [1]. *Phlebotomus sergenti* is the primary vector of anthroponotic CL (ACL), and *Ph. papatasi* is the main vector of zoonotic CL (ZCL) in Iran. ACL is endemic in some regions of Iran where it is considered to be anthroponotic, with the route of transmission from human to human [2, 3]. Most cases of CL in Iran are ZCL, which is endemic in many rural areas of 18 out of 31 provinces in the country, thus representing a significant public health problem [4–6]. VL, or kala-azar as it is referred to locally, has a very high mortality rate in the absence of timely diagnosis and treatment. VL is endemic in some foci of the country, including the provinces of North Khorasan, East Azerbaijan, Ardabil, Fars, Qom, Bushehr and Kerman. *Leishmania infantum* and *Leishmania donovani* have been reported using parasitological and molecular techniques [7, 8]. Three species of sand flies in southern Iran, namely *Phlebotomus major*, *Phlebotomus keshishiani* and *Phlebotomus alexandri*, and three species in the northwest and northeast regions of the country, namely *Phlebotomus kandelakii*, *Phlebotomus transcucasicus perfiliewi* and *Phlebotomus tobbi*, are reported to be the vectors of the disease [9]. In the southeast of Iran, massive spraying during the malaria eradication era resulted not only in a significant reduction in the number of malaria vectors in endemic areas, but also those for leishmaniasis . However, the annual incidence of leishmaniasis is increasing, and active foci of the disease are observed in both smal and large cities of Iran [10].

Several control methods are available to control sand flies, with an emphasis on insecticides. Due to the inaccessibility of the larval habitats of sand flies, it is impossible to control their larvae, so researchers have focused their efforts on the control of the adult sand flies. One of the approaches used is the application of chemical methods, such as residual spraying of indoor places and insecticide-impregnated mosquito nets [11, 12]. In some parts of the world, cases of resistance or tolerance of sand flies to insecticides have been reported [11]. In Iran, residual spraying with dichlorodiphenyltrichloroethane (DDT) against malaria vectors began in 1947 in most of the malaria-endemic areas. In 1957, insecticide resistance against DDT was reported in *Anopheles stephensi* [13, 14]. Rashti et al. investigated the susceptibility of *Ph. papatasi* to DDT in different agricultural fields of Iran between 1985 and 1988, and their results showed that sand flies in some areas of Isfahan Province had developed more tolerance to this insecticide [15]. Yaghoobi-Ershadi et al. subsequently confirmed this tolerance to DDT in Isfahan Province [4]. In 2012, Saeidi et al. evaluated and reported the level of susceptibility of *Ph. papatasi* species in the Badrood region of Isfahan Province to DDT and pyrethroids [14]. Aghaei Afshar et al. in 2011 declared that *Ph. papatasi* and *Ph. sergenti* were susceptible to DDT and deltamethrin in the Dehbakri area of Bam City [15]. Due to the lack of complete information on the susceptibility of sand flies in endemic areas of leishmaniasis to insecticides at the differential concentrations specified by WHO, which are recommended for periodic monitoring of insecticide resistance, we planned the present study. The objective was to survey current susceptibility levels of *Ph. sergenti*, the vector of urban leishmaniasis, *Ph. papatasi*, the vector of rural leishmaniasis and *Ph. alexandri*, the vector of VL, to the insecticides deltamethrin 0.05%, malathion 5% and DDT 4%, in wild sand fly populations during the season of highest activity in different foci of these diseases in Kerman province in 2019. Using the results of such studies, the WHO can develop specific guidelines for the control of sand flies, and such guidelines can be used to study and monitor resistance to insecticides, with the aim to implement measures to combat vectors in countries, including Iran.

## Methods

### Study areas

**Fig. 1:**
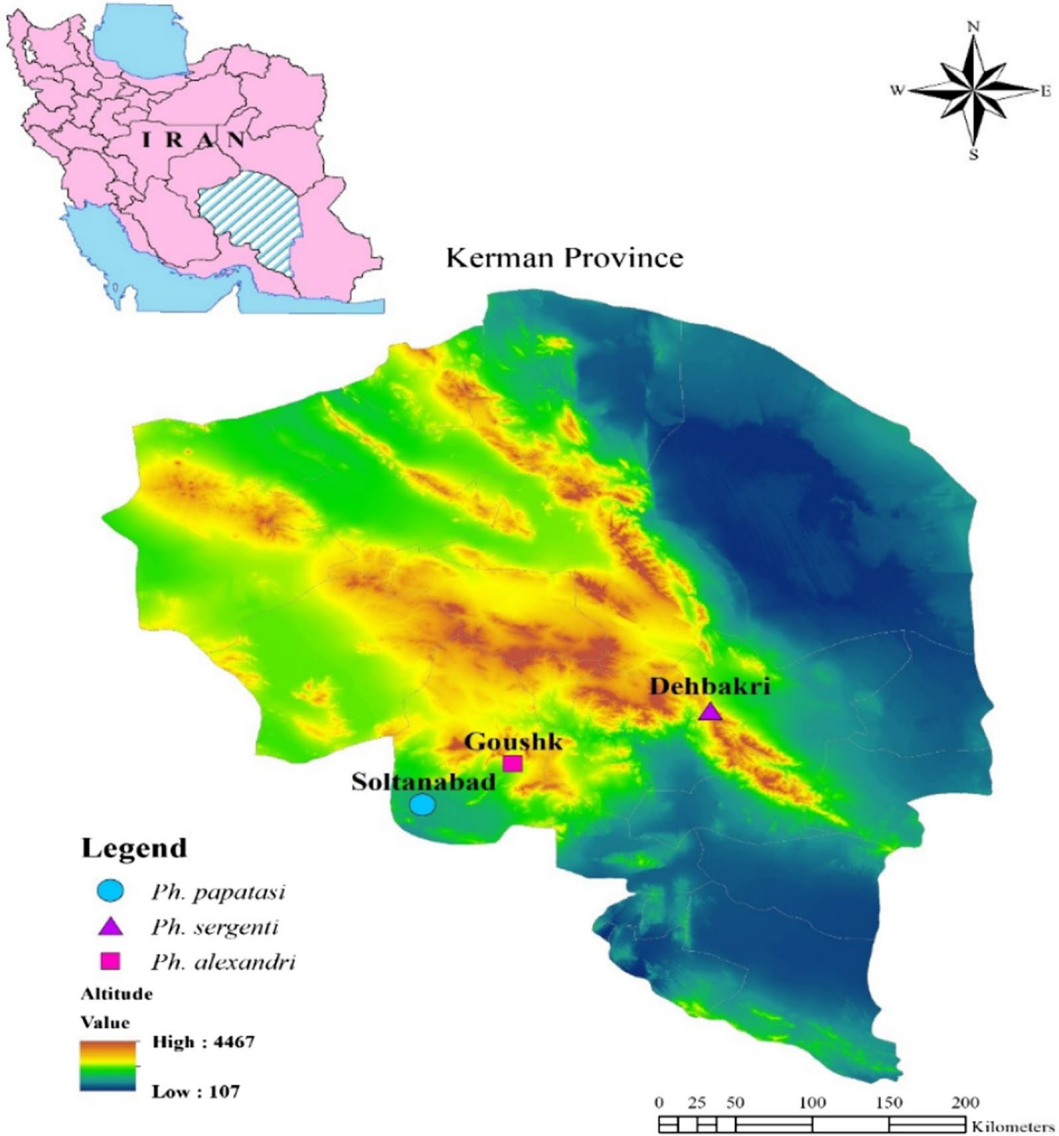
Map of Iran showing the sites of sand fly collection in Kerman Province

To evaluate the susceptibility of sand flies in different foci of Kerman province, we chose Bam district as the focus of ACL, Orzouieh district (Soltan Abad) as the focus of ZCL and the south of Baft district (Goushk) as the focus of VL [3, 16]. Bam district is geographically located in the southeast of Kerman Province and has a dry climate; Dehbakri, a small town surrounded by villages, was selected for sampling. Baft district is located in the southwest of Kerman Province, southeastern Iran, and has a cold climatic zone. Arzooieh district is located 125 km south of Baft district and has a hot and relatively humid climate (Fig. 1).

### Sand fly collection

Sand flies were collected by manual aspirator at different time intervals in study areas (Fig. 1) during the spring and summer of 2019. The collected live sand flies were transferred to cages kept under a wet towel. Adults were fed with 10% sucrose solution soaked on cotton pads, and the cages were transported to the laboratory at Kerman Leishmaniasis Research Center where the sand flies were maintained in the insectary at 27 ± 2 °C, 60 ± 10% relative humidity and a photoperiod of 14:10 h (light: dark).

A 10% sucrose solution was provided during the recovery period.

### Insecticides and susceptibility tests

Studies were conducted with the following insecticides: DDT 4.0% (batch number DD 265; expiry date: July 2022), deltamethrin 0.05% (batch number DE 381; expiry date: August 2019) and malathion 5.0% (batch number MA 234; expiry date: July 2020). The WHO provided the insecticide-impregnated test papers, and all susceptibility tests were conducted according to WHO tube-test guidelines [17].

During the tests, the wild sand flies were transferred into the holding tube, which was marked with a green dot. The exposure tubes were marked with a red dot and were lined with insecticide-impregnated test paper for different time durations (1.75, 3.5, 7, 15, 30 and 60 min). The holding tubes were transferred to the insectary for 24 h at 28 ± 2 °C, a photoperiod of 12:12 h (light:dark) and 75 ± 5% relative humidity. During the holding time, the sand flies were supplied with 20% fresh sugar solution on cotton pads. The mortality was recorded after a 24-h recovery period. Abbott’s correction formula was used to correct all mortalities compared to the control results (between 5 and 20%) [18]. The bioassay tests with a control mortality rate of > 20% were repeated. After each test, all sand flies (live and dead) were stored in 70% alcohol and subsequently mounted in a drop of Puri’s medium and identified by their morphological characteristics using a standard taxonomic key [19].

### Statistical analysis

The median lethal time causing 50% mortality (LT_50_) and median lethal time causing 90% mortality (LT_90_) of sand flies, regression equation and chi-square values were determined by probit analysis (Finney’s method) [20]. Data analysis was performed using SPSS software version 20 (SPSS IBM Corp., Armonk, NY, USA). The graphs were designed with Excel 2016 (Microsoft Corp., Redmond, WA, USA).

## Results

**Fig. 2:**
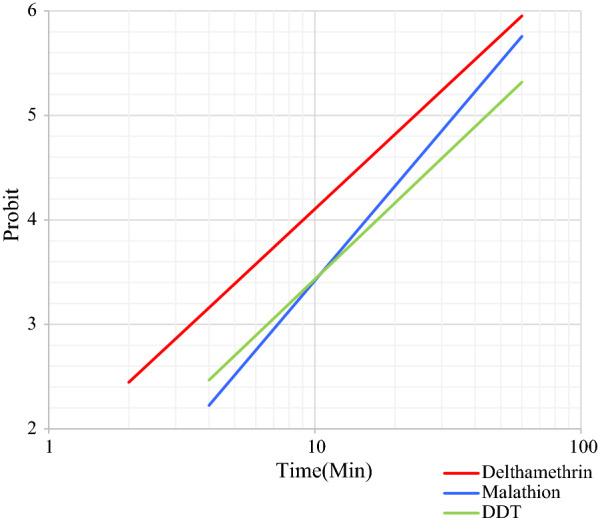
Comparison of regression lines, equations, and LT_50_ of *Phebotomus papatasi* exposed to DDT (4%), malathion (5%), and deltamethrin (0.05%). Abbreviations: DDT, Dichlorodiphenyltrichloroethane; LT_50_, lethal time causing 50% mortality

**Fig. 3:**
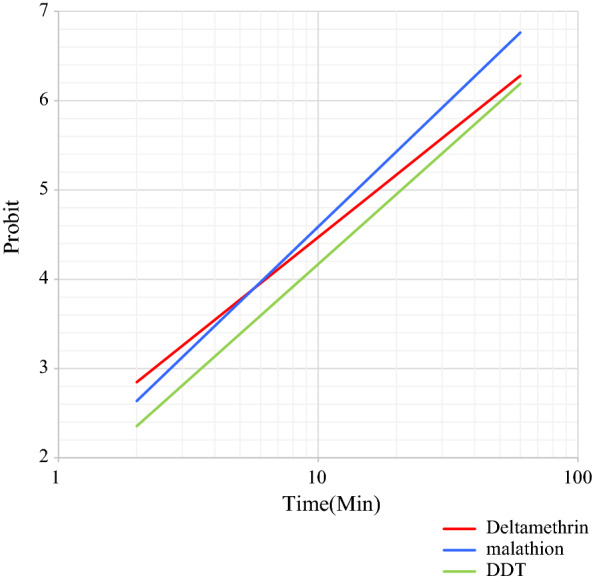
Comparison of regression lines, equations and LT_50_ of *Phebotomus alexandri* exposed to DDT (4%), malathion (5%) and deltamethrin (0.05%)

**Fig. 4:**
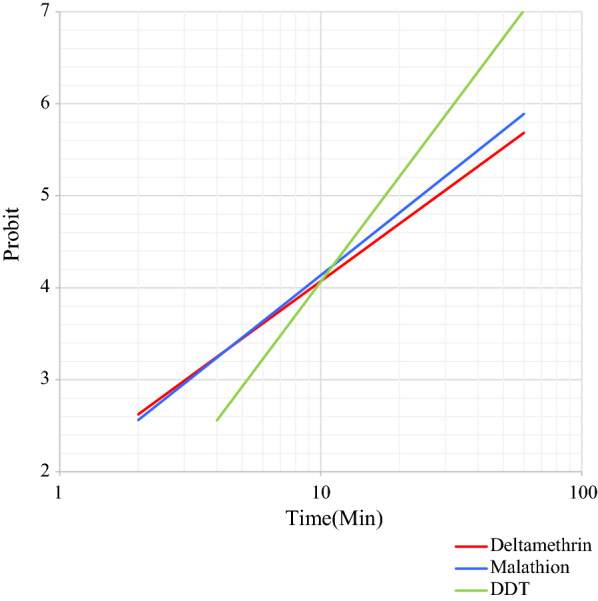
Comparison of regression lines, equations, and LT_50_ of *Phebotomus sergenti* exposed to DDT (4%), malathion (5%) and deltamethrin (0.05%)

The susceptibility bioassay tests on wild specimens of 25 blood-fed females of *Ph. papatasi*, *Ph. sergenti* and *Ph. alexandri* sand flies collected at the study sites in Kerman province were performed with different insecticide-impregnated papers, as shown in Tables 1, 2 and 3. The LT_50_ and LT_90_ values with their respective 95% CI for each species exposed to insecticides are given. The regression lines for mortality of these three sand fly species exposed to the three insecticides used are plotted against exposure times. Figures 2,  3 and 4, show the regression lines for each species separately when exposed to the three insecticides. All wild specimens died within 60 min of exposure to DDT 4%, malathion 5% and deltamethrin 0.05%; however, the mortality rate for *Ph. papatasi* following exposure to malathion and DDT was 91.6% and 66.6%, respectively.

**Table 1:** Parameters of probit regression lines of different insecticides against females of *Phlebotomus papatasi* in Kerman Province, Iran

Insecticide	A^a^	B (± SE)^b^	LT_50_ (min)^c^	LT_90_ (min)^c^	*χ*^2^ (*df*)^d^	*P-*value^e^
DDT	− 3.99	2.42 ± 0.68	44.33	149.75	1.34 (3)	> 0.05
Malathion	− 4.58	3.0 ± 0.74	33.61	89.80	4.96 (3)	> 0.05
Deltamethrin	− 3.27	2.37 ± 0.51	23.84	82.6	6.16 (4)	> 0.05

* DDT* dichlorodiphenyltrichloroethane,* SE* standard error

^a^A: *y*-intercept

^b^ B: Slope of the line

^c^LT_50_, LT_90_: Lethal time causing 50% mortality and 90% mortality, respectively

^d^*χ*^2^: Heterogeneity around the regression line

^e^*P*-value represents heterogeneity in the population of tested specimens

**Table 2:** Parameters of probit regression lines of different insecticides against females of *Phebotomus sergenti* in Kerman Province, Iran

Insecticide	A	B (± SE)	LT_50_ (min)	LT_90_ (min)	*χ*^2^ (*df*)	*P*-value
DDT	− 4.72	3.79 ± 0.8	17.64	38.43	1.55 (3)	> 0.05
Malathion	− 3.12	2.25 ± 0.49	24.17	89.59	7.35 (4)	> 0.05
Deltamethrin	− 3.0	2.07 ± 0.81	28.08	116.8	11.83 (4)	< 0.05

See footnotes to Table 1 for explanation of variables and abbreviations

**Table 3:** Parameters of probit regression lines of different insecticides against female of *Phebotomus alexandri* in Kerman Province, Iran

Insecticide	A	B (± SE)	LT_50_ (min)^c^	LT_90_ (min)^c^	*χ*^2^ (*df*)	*P*-value
DDT	− 3.43	2.6 ± 0.53	20.87	65	4.25 (4)	> 0.05
Malathion	− 3.2	2.79 ± 0.53	14.03	40.36	2.2 (4)	> 0.05
Deltamethrin	− 2.85	2.32 ± 0.46	16.89	60.16	4.07 (4)	> 0.05

See footnotes to Table 1 for explanation of variables and abbreviations

## Discussion

In order to plan for spraying endemic areas of leishmaniasis for control of sand flies and to consider the use of effective pesticides to protect the environment, the extent of insecticide susceptibility and resistance in sand fly vectors should be periodically reviewed. Because there are no available test procedures for monitoring insecticide resistance in sand flies, in the present study we used WHO procedures for malaria vectors. In accordance with the WHO guideline, we classified the bioassay results into three resistance classes: (i) 98–100% mortality, indicating susceptibility; (ii) 90–97% mortality, indicating resistance candidate and that more investigation is needed to confirm resistance; and (iii) mortality < 90%, indicating resistance [17].

Based on the results of the present study, *Ph. sergenti* and *Ph. alexandri* in the study areas are highly susceptible to the three insecticides tested. However, *Ph. papatasi* was found to be susceptible to deltamethrin (100% mortality), possibly resistant or tolerant to malathion (91.6% mortality) and resistant to DDT (66.6% mortality), with a higher susceptibility to malathion than to deltamethrin and DDT. *Phlebotomus sergenti*, although susceptible to all three insecticides tested, was more sensitive to DDT than to malathion and deltamethrin. In comparison, *Ph. alexandri* needed more time than *Ph. sergenti* to be killed at the same concentration of DDT in the study area at the LT_50_ level. Nevertheless, *Ph. alexandri* was found to be more susceptible to malathion and deltamethrin than *Ph. sergenti* and *Ph. papatasi*; *Ph. sergenti* was found to be more susceptible to malathion than *Ph. papatasi* to malathion; and *Ph. papatasi* was more susceptible to deltamethrin than *Ph. sergenti*.

Since the 1950s, malaria has been endemic in the south of Kerman province, and DDT and malathion have been employed in indoor residual spraying programs to combat the disease [13]. Sand flies have developed resistance to DDT and other pesticides due to spraying against malaria vectors [21].

In Iran, tolerance (existence resistance) of *Ph. papatasi* to DDT has been reported in the sand fly populations from Isfahan province [3]; however, this result showed that *Ph. papatasi* from Isfahan was more tolerant to DDT than *Ph. papatasi* from our study.  Surveying the susceptibility status of *Ph. papatasi* showed that this species is also resistant to DDT in CL foci in western regions of Iran [22]. Moreover, resistance to DDT was reported in *Ph. sergenti* in the endemic focus of CL in northern Iran [23]. Also, it seems that *Ph. kandelakii *and *Ph. perfiliewi* are possibly resistant to DDT in the VL endemic foci in northwestern Iran [24]. There have been reports of insecticide resistance in phlebotomine sand flies in a number of parts of the world; for example resistance to DDT was reported for *Ph. papatasi* in India [25] and for *Ph. argentipes* in India and Nepal [26]. The emergence of resistance to DDT was also shown in *Sergentomyia shorttii* in India [27]. In the present study, 91.6% mortality  indicated possible resistance to malathion in *Ph. papatasi*, similar to the possible resistance to malathion reported in *Ph. kandelakii *and *Ph. perfiliewi* in northwestern parts of Iran [24].

Leishmaniasis has long been present in the area, and insecticides have been employed to combat the disease’s vectors [28]. After the devastating earthquake in this area, insecticides have significantly been the main reason for the emergence of resistant species. Moreover, surveys of *Ph. papatasi* in the western parts of Iran showed that this species is possibly resistant to deltamethrin, permethrin and bendiocarb [22]. Given that there is no guideline on susceptibility tests in phlebotomine sand flies, the results of our study, based on guidelines for malaria vectors, indicate that more investigation is needed to confirm resistance in those species with < 90% mortality against a pesticide in our study.

## Conclusion

The findings of the present study indicate that resistance to DDT is emerging in *Ph. papatasi*, which could be an important factor for any future vector control program in the study area, as evidenced by several reports on different aspects of leishmaniasis in the country [28–32]. Our results provide a guideline for the Ministry of Health to control disease in different parts of the country.

## Data Availability

The datasets used and/or analyzed during the current study are available from the corresponding author on reasonable request.
